# Metabolomic and lipidomic assessment of the metabolic syndrome in Dutch middle-aged individuals reveals novel biological signatures separating health and disease

**DOI:** 10.1007/s11306-019-1484-7

**Published:** 2019-02-12

**Authors:** Izabella Surowiec, Raymond Noordam, Kate Bennett, Marian Beekman, P. Eline Slagboom, Torbjörn Lundstedt, Diana van Heemst

**Affiliations:** 1 AcureOmics AB, Umeå, Sweden; 2 Department of Internal Medicine, Section of Gerontology and Geriatrics, Leiden University Medical Center, PO Box 9600, 2300 RC Leiden, The Netherlands; 3 Department of Medical Statistics and Bioinformatics, Section of Molecular Epidemiology, Leiden University Medical Center, Leiden, The Netherlands

**Keywords:** Metabolic syndrome, Metabolomics, Lipidomics, Epidemiology

## Abstract

**Background:**

We aimed to identify novel metabolite and lipid signatures connected with the metabolic syndrome in a Dutch middle-aged population.

**Methods:**

115 individuals with a metabolic syndrome score ranging from 0 to 5 [50 cases of the metabolic syndrome (score ≥ 3) and 65 controls] were enrolled from the Leiden Longevity Study, and LC/GC–MS metabolomics and lipidomics profiling were performed on fasting plasma samples. Data were analysed with principal component analysis and orthogonal projections to latent structures (OPLS) to study metabolite/lipid signatures associated with the metabolic syndrome. In addition, univariate analyses were done with linear regression, adjusted for age and sex, for the study of individual metabolites/lipids in relation to the metabolic syndrome.

**Results:**

Data was available on 103 metabolites and 223 lipids. In the OPLS model with metabolic syndrome score (Y-variable), 9 metabolites were negatively correlated and 26 metabolites (mostly acylcarnitines, amino acids and keto acids) were positively correlated with the metabolic syndrome score. In addition, a total of 100 lipids (mainly triacylglycerides) were positively correlated and 10 lipids from different lipid classes were negatively correlated with the metabolic syndrome score. In the univariate analyses, the metabolic syndrome (score) was associated with multiple individual metabolites (e.g., valeryl carnitine, pyruvic acid, lactic acid, alanine) and lipids [e.g., diglyceride(34:1), diglyceride(36:2)].

**Conclusion:**

In this first study on metabolomics/lipidomics of the metabolic syndrome, we identified multiple novel metabolite and lipid signatures, from different chemical classes, that were connected to the metabolic syndrome and are of interest to cardiometabolic disease biology.

**Electronic supplementary material:**

The online version of this article (10.1007/s11306-019-1484-7) contains supplementary material, which is available to authorized users.

## Introduction

The metabolic syndrome is a strong risk factor for cardiovascular disease, and increases the risk of (cardiovascular) mortality (Isomaa et al. 2001; Lakka et al. 2002). The metabolic syndrome is a composite of metabolic disturbances in lipid (triglycerides and HDL cholesterol) and glucose metabolism, blood pressure regulation and being overweight (Grundy et al. 2005). The relative contribution of the different components to the diagnosis of the metabolic syndrome has changed during the past decades, owing to improved medication management and increased obesity prevalence (Afshin et al. 2017; Beltran-Sanchez et al. 2013). Importantly, four out of the five components of the metabolic syndrome (with the exception of HDL cholesterol) are causally associated with the risk of developing cardiovascular disease, as observed in Mendelian Randomization studies (Dale et al. 2017; Holmes et al. 2015; Lyall et al. 2017).

Besides the use of clinical markers, an increasing number of cohort studies uses metabolomics for the discovery of disease-related diagnostic and prognostic markers, as well as for an enhanced understanding of disease aetiology. For example, in several prospective cohort studies multiple metabolites were observed to be predictive for cardiovascular disease and mortality (Fischer et al. 2014; Wurtz et al. 2015). Studies on cardiometabolic disease phenotypes, however, have been generally focussed on the specific components of the metabolic syndrome (most notably glucose regulation and adiposity), not on the overall metabolic syndrome definition. With respect to the glucose component of the metabolic syndrome, different metabolites (e.g., glycerol, ketone bodies and branched-chain amino acids) have been identified in relation to (future) insulin resistance and incident type 2 diabetes mellitus (Mahendran et al. 2013a, b; Tillin et al. 2015; Wurtz et al. 2013, 2012b). Furthermore, the metabolite 1,5-anhydroglucitol has been identified as a novel risk factor for the development of type 2 diabetes, and a marker for short-term glycaemic control (Mook-Kanamori et al. 2014). Increased adiposity has been reported to cause changes in concentrations of multiple metabolites and lipids, which include fatty acids, ketone bodies and amino acids (Wurtz et al. 2014). However, these studies generally focused on a single component of the metabolic syndrome and investigated a limited number of metabolites. To the best of our knowledge, only 1 study examined the association between concentrations of several amino acids and the metabolic syndrome (Ntzouvani et al. 2017). The assessment of the heterogeneous population of metabolic syndrome patients could potentially highlight a common biochemical mechanism of importance for multiple cardiometabolic diseases.

A comprehensive approach, focusing on all components of the metabolic syndrome and including multiple metabolites and lipids from different chemical classes not often investigated in epidemiological cohort studies before, is likely to provide novel insights in cardiometabolic disease biology that facilitates in the search for novel innovative strategies for the treatment and prevention of cardiometabolic disease. In the presented study we aimed to identify metabolite and lipid patterns associated with the metabolic syndrome in middle-aged individuals as well as with the subcomponents of the metabolic syndrome in order to increase our understanding about the underlying biochemical processes.

## Methods

### Study setting and design

The present study was embedded in the Leiden Longevity Study, which aims to investigate biomarkers associated with familial longevity and healthy ageing. A more detailed description of the study design and recruitment strategy has been described elsewhere (Schoenmaker et al. 2006). In short, between 2003 and 2006 a total of 421 long-lived families were recruited, without selection based on health condition or demographics. Families were included when at least two long-lived siblings were still alive and fulfilled the age criteria of being at least 89 years for men and 91 years for women. Of these long-lived families, we recruited 1671 of their offspring and 744 partners thereof as controls resembling the general Dutch population at middle age. The Leiden Longevity Study was approved by the medical ethics committee of the Leiden University Medical Center. All participants provided written informed consent.

For the present study, we used fasting blood samples collected between 2006 and 2008 from a subpopulation (N = 280) of the Leiden Longevity Study that lived in close approximation (< 45 min by car) from the research center, as we have previously described (Rozing et al. 2010). Within this subpopulation, cases of the metabolic syndrome were identified on the basis of the criteria from the Third Report of the National Cholesterol Education Program (Klose et al. 2014), which is dependent on 5 subcomponents (waist circumference > 102 cm in men, > 88 cm in women; triglyceride concentration ≥ 1.69 mmol/L; HDL cholesterol (HDL-C) < 1.04 mmol/L in men, < 1.29 mmol/L in women; fasting glucose ≥ 6.1 mmol/L or diagnosed diabetes; systolic blood pressure ≥ 130 mmHg, diastolic blood pressure ≥ 85 mmHg, or treated for hypertension) giving a score ranging from 0 to 5 points. Using this score, participants with a score ≥ 3 were considered as having the metabolic syndrome; others were considered as controls without the metabolic syndrome.

For the present study, for each of the sample subclasses (N = 24, based on metabolic syndrome score, sex and offspring/control group), multivariate characterization was used for the design of experiment-based sample selection, as was described before (Surowiec et al. 2017a, b). In short, for each metabolic syndrome score value, two-component PCA models on available clinical data were constructed for four main classes of samples; a full two-factor, two-level factorial design with one centre point was fitted to the PCA score plots, aiming for the selection of five samples for each subclass (offspring and controls; stratified by sex), and hence 20 samples from each metabolic syndrome score value (ranging from 0 to 5). It was however not possible to fully follow the presented strategy for all groups, either because of low number of samples for specific groups (for example for metabolic syndrome score equal 5), or because of not even distribution of the samples on the PCA score plots. In the last case, to obtain a balanced and representative selection, additional samples were included in the study. At the end, 115 representative samples were chosen, with 17, 25, 23, 23, 22 and 5 samples for the metabolic syndrome score equal 0, 1, 2, 3, 4 and 5 respectively. If possible, we did not include samples from participants who were on antihypertensive or lipid-lowering medication (a total of 31 users of antihypertensive and 18 users of lipid-lowering medication remained in the analyses).

### Anthropometrics and clinical information

Waist circumference was measured halfway between the lower costal margin and the iliac crest with participants in a standing position. Systolic and diastolic blood pressure were measured in resting condition twice; the average thereof was used for the analyses. Diagnosis of hypertension was based on systolic and diastolic blood pressure as well as on the use of antihypertensive medication. Use of antihypertensive medication was retrieved from the pharmacist of the participant. Diagnosis of diabetes mellitus was based on a fasting blood glucose concentration > 6.9 mmol/L, a diagnosis by a medical specialist (by questionnaire from the general practitioner) or by the use of glucose-lowering medication (by questionnaire from the pharmacist).

All routine clinical serum measurements were performed using fully automated equipment and standardized protocols. Glucose, Hb1Ac, high-sensitivity C-reactive protein, HDL-C and triglyceride concentrations were measured with the Hitachi Modular P800 (Roche, Almere, the Netherlands). Alanine transaminase (ALT), aspartate aminotransferase (AST) and gamma-glutamyltransferase (GGT) concentrations were measured on an Abbott ci8200 (Roche, Almere, the Netherlands). ALT and AST were measured using the NADH (with P-5′-P) methodology and GGT by measuring the substrate l-gamma-glutamyl-3-carboxy-4-nitroanilide methodology. Coefficients of variation of all measures were below 5%.

Information on alcohol intake and current smoking status were retrieved by questionnaire. Information on total caloric intake was retrieved via a validated food frequency questionnaire (Verkleij-Hagoort et al. 2007).

The anthropometric and clinical characteristics of the participants were provided for cases of the metabolic syndrome (metabolic syndrome score ≥ 3) and controls separately as means (with standard deviation) or numbers (percentage) (Table 1).

**Table 1:** Characteristics of the study population

	Metabolic syndrome (N = 50)	Controls (N = 65)
Age in years, mean (SD)	64.4 (6.1)	62.0 (6.5)
Men, N (%)	26 (52.0)	34 (52.3)
Alcohol intake in glasses/week, number (IQR)	6.0 (0.0, 14.0)	9.0 (3.0, 14.0)
Current smoking, N (%)	3 (6.0)	9 (13.8)
Total caloric intake in kCal/day, median (IQR)^a^	1774 (1498, 2053)	1987 (1656, 2491)
Waist circumference in cm, mean (SD)	106.4 (10.3)	95.9 (11.7)
Triglyceride concentration in mmol/L, mean (SD)	2.34 (1.29)	1.17 (0.53)
HDL cholesterol in mmol/L, mean (SD)	1.13 (0.30)	1.59 (0.41)
Glucose in mmol/L, mean (SD)	6.9 (3.1)	5.4 (1.3)
Type 2 diabetes mellitus, N (%)	11 (22.0)	5 (7.7)
Systolic blood pressure in mmHg, mean (SD)	147.3 (17.8)	129.7 (18.0)
Diastolic blood pressure in mmHg, mean (SD)	84.6 (9.1)	76.3 (8.8)
Use of antihypertensive agents, N (%)	26 (52.0)	5 (7.7)
*Metabolic syndrome components (according to cut offs)*		
Waist circumference, N (%)	42 (84.0)	24 (36.9)
Triglycerides, N (%)	35 (70.0)	7 (10.8)
HDL cholesterol, N (%)	31 (62.0)	2 (3.1)
Glucose, N (%)	28 (56.0)	10 (15.4)
Blood pressure, N (%)	46 (92.0)	28 (43.1)
HbA1c in %, mean (SD)^b^	5.8 (1.3)	5.2 (0.6)
High-sensitivity C-reactive protein in mg/dL, median (IQR)	1.9 (0.6, 3.5)	1.0 (0.6, 1.9)
Alanine transaminase in U/L, median (IQR)	18.0 (14.0, 23.5)	16.0 (12.0, 18.5)
Aspartate transaminase in U/L, median (IQR)	22.0 (17.0, 28.0)	19.0 (17.5, 23.5)
Gamma-glutamyltransferase in U/L, median (IQR)	27.0 (19.8, 53.0)	22.0 (13.0, 36.5)

*HDL* high-density lipoprotein, *IQR* interquartile range, *N* number of participants, *SD* standard deviation

^a^Assessed in 42 metabolic syndrome cases and 49 control participants

^b^Measured in 42 metabolic syndrome cases and 56 control participants.

### Metabolomics analyses

Fasting EDTA plasma samples from the participants, which were not thawed before, were thawed on ice; 630 µL of extraction mixture (H_2_O:methanol (1:9, v/v)) was added to 70 µL of plasma. Extraction of the metabolites from the sample was then carried out using a MM301 vibration Mill (Retsch GmbH & Co. KG, Haan, Germany) at a frequency of 30 Hz for 2 min. Samples were stored on ice for 2 h to allow protein precipitation, after which they were centrifuged at 18 620 RCF for 10 min at 4 °C. An aliquot (200 µL) of the resulting supernatant was transferred to a liquid chromatography vial and evaporated to dryness at room temperature in a miVac QUATTRO concentrator (Genevac LTD, Ipswich, UK). Subsequently, samples were dissolved in 20 µL of methanol:water (1:1 ratio) mixture and analysed with liquid chromatography-mass spectrometry (LC–MS) system as described in detail in Supplementary Methods. Gas chromatography-mass spectrometry (GC–MS) analyses was performed after metabolite derivatization as described before (Jiye et al. 2005); a detailed description on the methodology is given in Supplementary Methods.

### Lipidomics analysis

Fasting plasma samples from the participants, which were not thawed before, were thawed on ice and 110 µL of extraction mixture (chloroform:methanol (2:1, V/V)) was added to 20 µL of plasma sample. Extraction was carried out using a MM301 vibration Mill (Retsch GmbH & Co. KG, Haan, Germany) at a frequency of 30 Hz for 2 min. Subsequently, samples were stored at ambient temperature for 60 min before being centrifuged at 18 620 RCF for 3 min at 4 °C. A 50 µL aliquot of the resulting lower phase was transferred to a LC vial, 70 µL of a chloroform:methanol (2:1, V/V) mixture were added and samples were briefly shaken before being analysed by LC–MS as described in detail in Supplementary Methods.

### Compound identification

Targeted feature extraction of the acquired LC–MS data was performed using the Profinder™ software package, version B.06.00 (Agilent Technologies Inc., Santa Clara, CA, USA) and an in-house retention-time based and mass-spectra based libraries consisting of 713 metabolites and 487 lipid species. These libraries contained compounds from chemical classes such as acylcarnitines, amino acids, carbohydrates, fatty acids, lysophosphatidylcholines, organic acids, phosphatidylcholines, sphingomyelins, triglycerides and others. Detection of the compounds was based on the following parameters: allowed ion species in positive ionization mode: (+H, +Na, +K, +NH_4_); in negative ionization mode: (–H, +HCOO); peak spacing tolerance: 0.0025–7 ppm; isotope model: common organic molecules; charge state: 1; mass tolerance: 10 ppm; retention time tolerance: 0.1 min. After extraction of the peaks, each compound was manually checked for mass and retention time agreement with appropriate standards from the library; peaks with bad characteristics (e.g., overloaded, sample noise, non-Gaussian) were excluded from the analysis. Identification of compounds was confirmed by comparison of MS/MS spectra with MS/MS spectra of relevant compounds from the library.

Non-processed files from GC–MS were exported in NetCDF format to a MATLAB-based in-house script where all data pre-treatment procedures such as baseline correction, chromatogram alignment, and peak deconvolution were performed. Metabolite identification, was implemented within the script and was based on the retention index (RI) values and MS spectra from the in-house mass spectra library established by the Swedish Metabolomics Centre (Umeå, Sweden) and consisting of 585 compounds [Level 1 identification according to the Metabolomics Standards Initiative (Salek et al. 2013)].

### Data processing and multivariate and univariate data analysis

For the LC–MS analysis of the metabolites, a combined dataset was used, with compounds included that could be detected in either negative or positive ion modes. In case a single metabolite was detected in both the negative and positive ion mode, the signal with the highest intensity was used for the statistical analyses. When metabolites were detected with both the LC–MS and GC–MS methodology, the signal detected with the GC–MS method was used for the statistical analyses. The LC–MS metabolite and lipid signals were normalized to the total peak area prior to further statistical analyses. GC–MS data were normalized to internal standards as described before (Redestig et al. 2009).

Metabolite and lipid data were imported separately into SIMCA software (version 14.0, Sartorius Stedim Biotech Umetrics AB, Umeå, Sweden) for multivariate analyses. All data were mean centred and scaled to unit variance. Principal component analysis (PCA) was used to obtain an overview of the variation in the data and to check for trends and potential outliers for cases of the metabolic syndrome and controls. Seven-fold cross-validation was used for calculating the models. Orthogonal partial least squares (OPLS) method was used to correlate metabolite and lipid profiles with the continuous metabolic syndrome score (Y variable) of the study participants; 1 + 0 or 1 + 1 component models were used to avoid possible over-fitting (Trygg et al. 2002). The significance of a metabolite for classification in the OPLS models was specified by calculating the 95% confidence interval for the loadings using the jackknife method, which attempts to find precision of an estimate, by iteratively making subsets in which estimates are calculated (Efron et al. 1983). OPLS models were also created for the separate subcomponents of the metabolic syndrome as the Y variable (waist circumference, plasma fasting triglycerides, HDL-C, and glucose concentrations, and systolic and diastolic blood pressure). Validity and degree of overfitting of the OPLS models was checked by conducting CV-ANOVA (ANalysis Of VAriance testing of Cross-Validated predictive residuals) and permutation analyses.

In addition, we conducted univariate analyses on the metabolites and lipids using linear regression in the R statistical environment. Metabolites and lipids were log-transformed and subsequently standardized to approximate a standard normal distribution (mean = 0, standard deviation = 1). Hence, results from the univariate analyses can be interpreted as the difference in standard deviation in metabolite/lipid level between cases of the metabolic syndrome and controls. Results were repeated with the metabolic syndrome score as a continuous determinant. Outlying metabolite and lipid levels (> 4 standard deviations from the mean) were excluded from the analyses. As we studied a high number of associations between exposure and metabolite/lipid, there is a risk of getting false-positive results. To correct for multiple testing, we first calculated the number of independent metabolites and lipids based on the methodology described by Li et al. (2005), and subsequently corrected our threshold for statistical significance accordingly. Univariate analyses were visualized using the ggplot2 package in the R statistical environment (Wickham 2009).

## Results

### Characteristics of the study population

According to the used clinical classification of the metabolic syndrome, samples were available for 50 cases (metabolic syndrome score ≥ 3) and 65 controls (Table 1). Both groups were similar with respect to age (64.4 [SD 6.1] versus 62.0 [SD 6.5] years, respectively) and percentage of men (52.0% versus 52.3%, respectively). Cases currently smoked less and had a lower alcohol intake and lower total caloric intake compared to controls. In line with the clinical classification of the metabolic syndrome, components of the metabolic syndrome were generally higher in cases compared to controls with the exception of HDL cholesterol. Furthermore, cases had a higher mean HbA1C and higher median hsCRP and had moderately higher median liver enzyme concentrations.

### Multivariate metabolite profiling

**Fig. 1:**
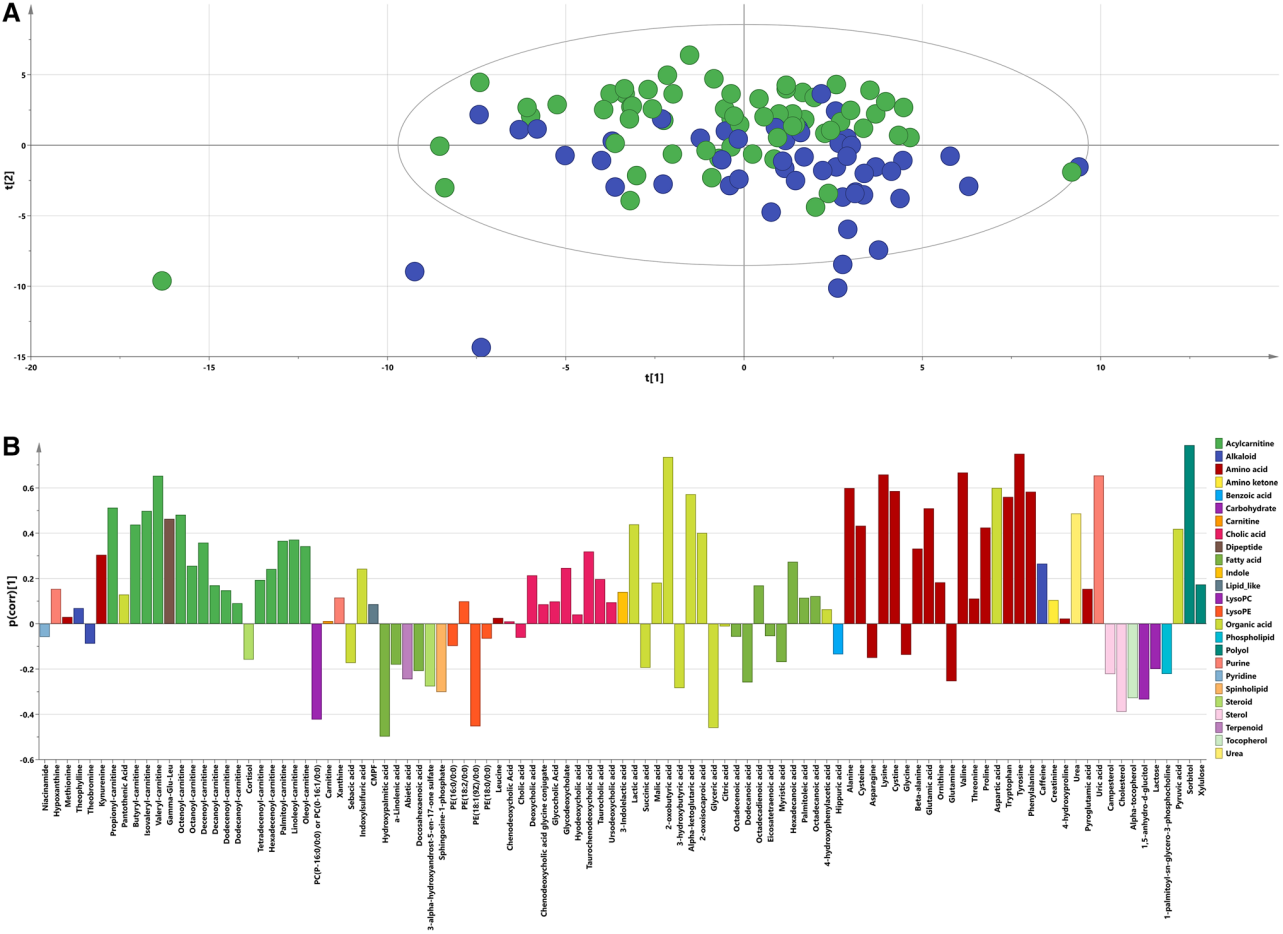
Metabolite profiling. **a** PCA score plot on metabolic data with samples colored according to their respective groups: blue dots signify individuals with the metabolic syndrome (metabolic syndrome score 3–5) and green dots individuals without metabolic syndrome (metabolic syndrome score 0–2); x axis – t[1] first score (R^2^X = 0.146), y axis—t[2], second score (R^2^X = 0.110). **b** Metabolite predictive loading vector (p(corr)) from the OPLS model with the metabolic syndrome score as the Y variable; metabolites are colored according to their chemical classes; p(corr) values indicate when a compound is positively (positive p(corr) value) or negatively (negative p(corr) value) correlated with the metabolic syndrome score

PCA on 115 samples and 103 metabolites resulted in a model with 7 components, which explained 53% of the total variation (R^2^X(cum) = 0.53), and identified 3 samples outside Hotelling’s T2 range that remained in the subsequent analyses (Fig. 1a). In the PCA plot, a trend was visible with samples from cases with metabolic syndrome (metabolic syndrome score ≥ 3) located more frequently in the lower half of the plot. A total of 11.7% of the total variation in the data was explained by the predictive component of the OPLS model with the metabolic syndrome score (ranging from 0 to 5) as the Y variable (1 + 0 model, Q^2^ = 0.39; R^2^X(cum) = 0.12; CV-ANOVA p-value: 1.2 × 10^−12^). Additional diagnoses by permutation analyses for the Y variable (Supplementary Fig. 1) showed Y-axis intercepts below 0.3 for R^2^Y and below 0.05 for Q^2^, indicating the OPLS model was not influenced by overfitting. The metabolic profile connected to the metabolic syndrome score (p(corr) vector from the OPLS model] is presented in Fig. 1b, and significant results are summarized in Table 2 (all results are summarized in Supplementary Table 1). In the metabolomics dataset, a total of 35 metabolites were significantly correlated to the metabolic syndrome score, based on jackknife confidence intervals; multiple amino acids, organic acids and acylcarnitines were positively correlated with the metabolic syndrome score and several compounds (e.g., some fatty acids and sterols) were negatively correlated with the metabolic syndrome score. When metabolic syndrome components were used as Y variables in the OPLS model, multiple metabolites were found to be significantly correlated with these components (Supplementary Table 1). Metabolic profiles for the different components (p(corr) vectors) were correlated to the metabolite profile connected to the metabolic syndrome score and strongest correlations with the metabolic syndrome score were found with systolic blood pressure (R^2^ = 0.96) and HDL-C (R^2^ = − 0.94), and lowest with glucose (R^2^ = 0.55).

**Table 2:** Metabolites connected to the metabolic syndrome score

	Chemical class	p(corr) vector
*Lower levels with higher metabolic syndrome score*		
PC(P-16:0/0:0) or PC(0–16:1/0:0)	LysoPC	− 0.42
Hydroxypalmitic acid	Fatty acid	− 0.53
2-Hydroxyhexadecanoic acid	Fatty acid	− 0.52
Sphingosine-1-phosphate	Spinholipid	− 0.32
PE(18:1(9Z)/0:0)	LysoPE	− 0.46
Glyceric acid	Organic acid	− 0.46
Campesterol	Sterol	− 0.23
Cholesterol	Sterol	− 0.39
1,5-Anhydro-d-glucitol	Carbohydrate	− 0.33
*Higher levels with higher metabolic syndrome score*		
Kynurenine	Amino acid	0.30
Butyryl-carnitine	Acylcarnitine	0.44
Isovaleryl-carnitine	Acylcarnitine	0.50
Valeryl-carnitine	Acylcarnitine	0.65
Gamma-Glu-Leu	Dipeptide	0.46
Indoxylsulfuric acid	Organic acid	0.24
Deoxycholic acid	Cholic acid	0.23
Lactic acid	Organic acid	0.44
2-Oxobutyric acid	Organic acid	0.73
Alpha-ketoglutaric acid	Organic acid	0.57
2-Oxoisocaproic acid	Organic acid	0.40
Alanine	Amino acid	0.60
Cysteine	Amino acid	0.43
Lysine	Amino acid	0.65
Cystine	Amino acid	0.58
Glutamic acid	Amino acid	0.51
Valine	Amino acid	0.67
Proline	Amino acid	0.43
Aspartic acid	Organic acid	0.60
Tryptophan	Amino acid	0.56
Tyrosine	Amino acid	0.75
Phenylalanine	Amino acid	0.58
Urea	Urea	0.49
Uric acid	Purine	0.65
Pyruvic acid	Organic acid	0.42
Sorbitol	Polyol	0.78

P(corr) values obtained from the OPLS model with metabolic syndrome score as Y variable. Statistical significance determined with jackknife confidence intervals

### Multivariate lipid profiling

**Fig. 2:**
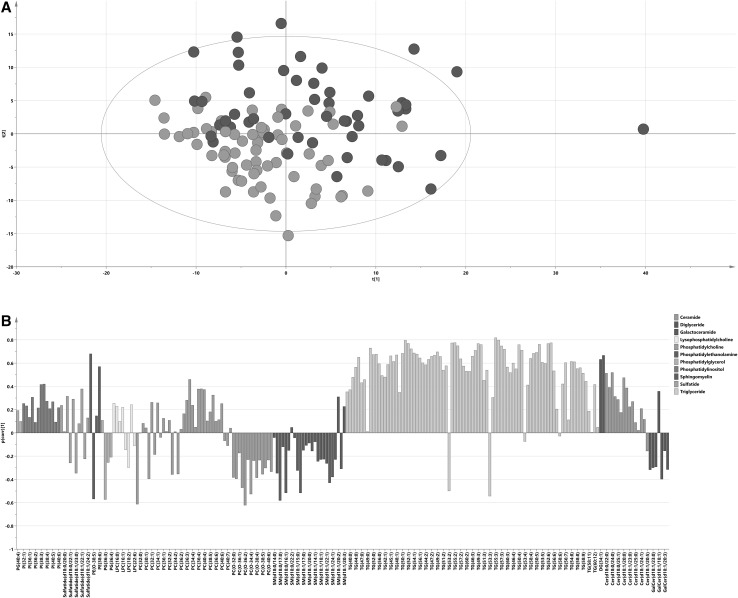
Lipid profiling. **a** PCA score plot on lipidomics data with samples colored according to their respective groups: blue dots signify individuals with the metabolic syndrome (metabolic syndrome score 3–5) and green dots individuals without metabolic syndrome (metabolic syndrome score 0–2); x axis – t[1] first score (R2X = 0.304), y axis – t[2], second score (R2X = 0.155). **b** Lipidomics predictive loading values (p(corr)) from the OPLS model with the metabolic syndrome score as the Y variable; metabolites are colored according to their chemical classes; p(corr) values indicate when a compound is positively (positive p(corr) value) or negatively (negative p(corr) value) correlated with the metabolic syndrome score

PCA on 115 samples and 223 lipids gave a model of 12 components explaining 83% of the total variation in the data (R^2^X(cum) = 0.83), and identified one sample outside Hotelling’s T2 range that remained in subsequent analyses (Fig. 2a). A trend was visible in the PCA plot with samples from individuals with the metabolic syndrome (metabolic syndrome score ≥ 3) being more frequently located on the upper half (positive t_2_ values) of the plot. A total of 19.4% of the total variation in the data was explained by the predictive component of the OPLS model with the metabolic syndrome score as Y (1 + 1 model, Q^2^ = 0.47, R^2^X(cum) = 0.453, CV-ANOVA p value: 1.4 × 10^−14^). Additional diagnosis by the permutation analyses for the Y variable (Supplementary Fig. 2) showed Y-axis intercepts below 0.3 for R^2^Y and below 0.05 for Q^2^, indicating the OPLS model was not influenced by overfitting. The lipidomic profile (p(corr) vector from the OPLS model) connected to the metabolic syndrome score in the OPLS model is presented in Fig. 2b and significant lipids are presented in Table 3 (complete list is summarized in Supplementary Table 2). A total of 110 lipids were significantly correlated to the metabolic syndrome score. Of these, 100 lipids were positively correlated (mainly triglycerides with 76 compounds, phosphatidylcholines, phosphatidylinositols and ceramides) with the metabolic syndrome score and 10 lipids were negatively correlated with the metabolic syndrome score.

**Table 3:** Lipids connected to the metabolic syndrome score

	Lipid class	p(corr) vector
*Lower levels with higher metabolic syndrome score*		
PE(O-38:5)	Phosphatidylethanolamine	− 0.57
PG(36:3)	Phosphatidylglycerol	− 0.57
PG(38:3)	Phosphatidylglycerol	− 0.26
PC(31:0)	Phosphatidylcholine	− 0.61
PC(O-34:2)	Phosphatidylcholine	− 0.47
PC(O-36:2)	Phosphatidylcholine	− 0.62
PC(O-38:7)	Phosphatidylcholine	− 0.33
SM(d18:0/17:0)	Sphingomyelin	− 0.58
TG(53:2)	Triglyceride	− 0.50
*Higher levels with higher metabolic syndrome score*		
PI(32:1)	Phosphatidylinositol	0.25
PI(34:2)	Phosphatidylinositol	0.31
PI(38:3)	Phosphatidylinositol	0.42
PI(36:4)	Phosphatidylinositol	0.42
Sulfatide(d18:0/18:0)	Sulfatide	0.24
Sulfatide(d18:0/22:0)	Sulfatide	0.32
Sulfatide(d18:1/22:1)	Sulfatide	0.38
PE(38:4)	Phosphatidylethanolamine	0.68
PE(38:6)	Phosphatidylethanolamine	0.57
PC(32:1)	Phosphatidylcholine	0.26
PC(38:3)	Phosphatidylcholine	0.46
PC(36:4)	Phosphatidylcholine	0.38
PC(38:4)	Phosphatidylcholine	0.38
PC(40:4)	Phosphatidylcholine	0.37
PC(40:5)	Phosphatidylcholine	0.33
PC(40:6)	Phosphatidylcholine	0.25
DG(34:1)	Diglyceride	0.63
DG(36:2)	Diglyceride	0.67
Cer(d18:0/22:0)	Ceramide	0.51
Cer(d18:0/23:0)	Ceramide	0.39
Cer(d18:0/24:0)	Ceramide	0.52
Cer(d18:1/20:0)	Ceramide	0.48
Cer(d18:1/22:0)	Ceramide	0.39
GalCer(d18:1/18:1)	Galactoceramide	0.36
PI(32:1)	Phosphatidylinositol	0.25
PI(34:2)	Phosphatidylinositol	0.31

P(corr) values obtained from the OPLS model with metabolic syndrome score as Y variable. Statistical significance determined with jackknife confidence intervals. Results for the triacylglycerides with positive p(corr) vectors are not presented

### Univariate metabolite and lipid analyses

**Fig. 3:**
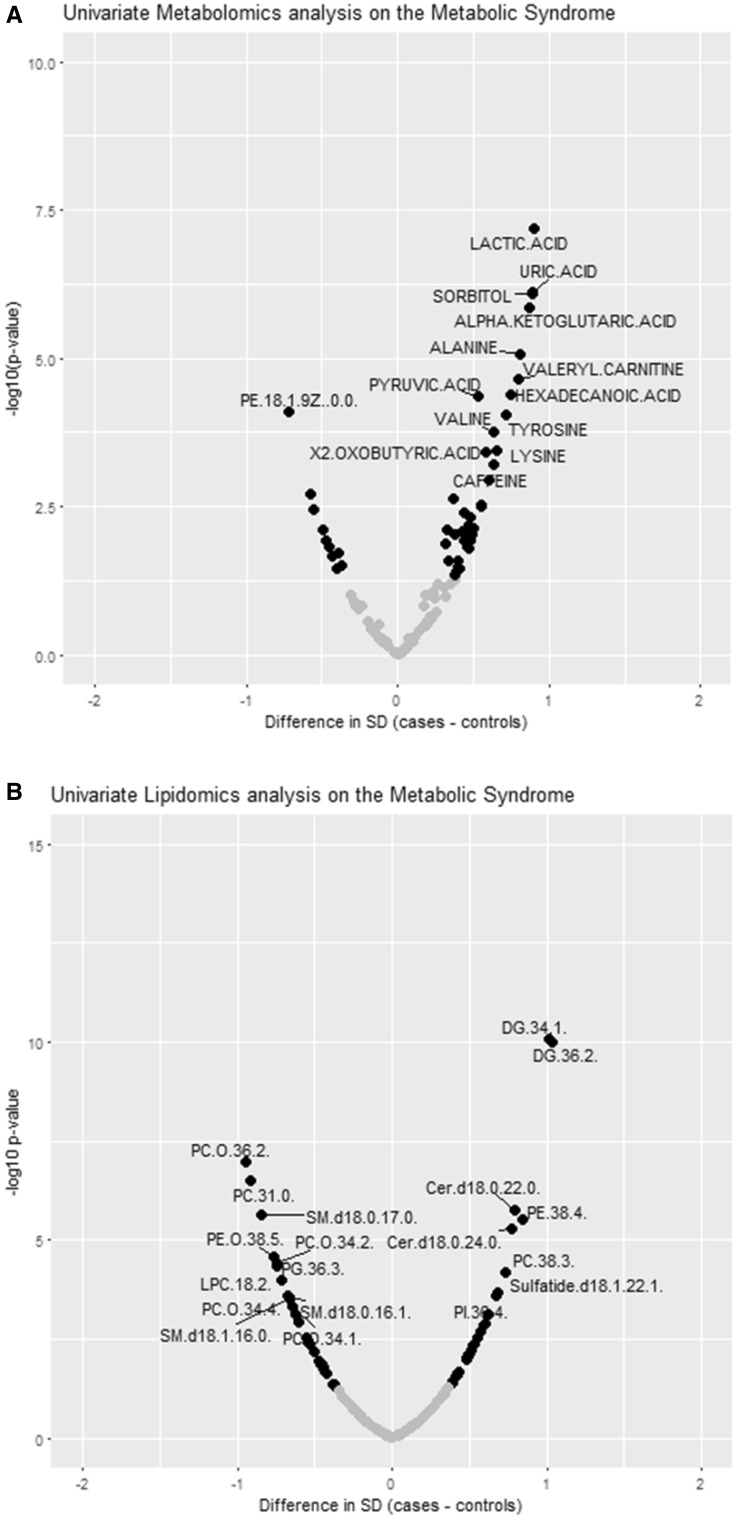
Univariate metabolite and lipids analyses. **a** Univariate metabolite analyses on the metabolic syndrome. **b** Univariate lipid analyses on the metabolic syndrome. Analyses can be interpreted as the difference in metabolite/lipid level in standard deviation in cases of the metabolic syndrome as compared to controls. The difference between cases of the metabolic syndrome (in standard deviation) is presented on the x-axis; the − log(p-value) of the comparison is presented on the y-axis. Metabolites/lipids that were labelled in the figures were those that remained significant after correction for multiple testing; compounds with a p-value < 0.05 are presented as solid black dots in the plot. In our dataset, there were 67 independent metabolites (p-value cut-off = 7.46 × 10^−4^) and 73 independent lipids (p-value cut-off = 6.85 × 10^−4^)

In our data, we had 67 independent metabolites and 73 independent lipids. Hence, we used a p-value threshold of 7.46 × 10^−4^ for the metabolite analyses and 6.85 × 10^−4^ for the lipid analyses. In the univariate regression analyses on standardized metabolite (Fig. 3a) and lipid (Fig. 3b) levels, where we compared metabolic syndrome cases and controls, we identified multiple metabolites and lipids that had higher (13 metabolites; 8 lipids) or lower (1 metabolite; 10 lipids) levels in cases of the metabolic syndrome after correction for multiple testing. Examples of metabolites associated with the metabolic syndrome were valeryl carnitine, pyruvic acid, lactic acid and alanine; examples of lipids associated with the metabolic syndrome were diglyceride(34:1) and diglyceride(36:2). Similar results were observed with the metabolic syndrome score as a continuous determinant in the analyses. Summary statistics are presented in Supplementary Table 3 (metabolites) and Supplementary Table 4 (lipids).

## Discussion

In the present study, which is the first of its kind, we observed multiple metabolites and lipids from different chemical classes to be connected to the metabolic syndrome that have not been often described before in epidemiological studies, which includes acylcarnitines and keto acids. Collectively, our findings highlight the role of multiple different biochemical pathways connected to the metabolic syndrome that could be used in the design of novel interventions for the treatment and prevention of cardiometabolic disease.

Our study replicates multiple observations from other studies on different cardiometabolic disease outcomes, which includes multiple amino acids, 1,5-anhydroglucitol and uric acid. Most notably, previous studies found associations between high concentrations of branched-chain amino acids and the risk of type 2 diabetes mellitus (Wurtz et al. 2012a, 2013) possibly by the disturbance of fatty acid metabolism in mitochondria (Newgard 2012). In our study population, valine (one of the branched-chain amino acids) was positively associated with the metabolic syndrome in both the OPLS model and univariate analysis. Interestingly, high levels of valine have been associated with increased oxidative stress and inflammation through the activation of mTOC1 (Zhenyukh et al. 2017). In addition, higher levels of many other amino acids were associated with the metabolic syndrome score as well, a result which is in line with the results from another cross-sectional study (Ntzouvani et al. 2017). Alanine, which had the strongest association in our univariate analyses, has previously been documented to directly affect beta-cell function and insulin secretion (Newsholme et al. 2005). Furthermore, in line with previous research on type 2 diabetes mellitus (Mook-Kanamori et al. 2014) and cardiovascular mortality in normoglycaemic individuals (Ouchi et al. 2017), metabolic syndrome cases had lower levels of carbohydrate 1,5-anhydroglucitol compared to controls. Most likely, this observation is explained by the diabetes subcomponent as reflected by a significant correlation with the glucose component in the OPLS model. Finally, in line with our research findings, though these associations were previously found not to be causal (Palmer et al. 2013; Sluijs et al. 2015), high levels of uric acid in serum have been associated with an increased risk of type 2 diabetes (Dehghan et al. 2008) as well as with hypertension, the metabolic syndrome and cardiovascular disease (Soltani et al. 2013). As the direction of effects of these metabolites went in the expected direction, our used platform and study design seem to be suitable for the identification of novel biochemical pathways.

A class of compounds which brought particular novel insights in the biochemical pathways related to the metabolic syndrome from our data are the acylcarnitines which were positively correlated with the metabolic syndrome. Specifically, we found valeryl-carnitine to show the strongest connection with the metabolic syndrome in the OPLS and univariate analyses. As not much has been described about this particular metabolite, future studies focussing on valeryl-carnitine in particular are required to elucidate its role in cardiometabolic disease. Acylcarnitines are required for the transport of fatty acids across the mitochondrial membrane for β-oxidation (Mihalik et al. 2010). Higher concentrations of acylcarnitines in blood have been associated with obesity, insulin resistance and type 2 diabetes mellitus in humans (Floegel et al. 2014; Gall et al. 2010; Mihalik et al. 2010; Pallares-Mendez et al. 2016). In one previous publication, higher acylcarnitine concentrations were shown to cause imbalances between insulin synthesis and insulin secretion, which consequently caused beta cell dysfunction in human and mouse pancreatic tissue samples (Aichler et al. 2017). In line, we found multiple acylcarnitines to be positively correlated with fasting glucose levels in the OPLS model.

Another main chemical class with strong positive correlations with the metabolic syndrome, as shown by OPLS and univariate analyses, were keto acids. For example alpha-ketoglutaric acid, lactic acid and pyruvic acid. Alpha-ketoglutaric acid, although not described in recent clinical studies, was found to affect TOR signalling, which affects insulin signalling, and has been associated with longevity in nematode worms (Chin et al. 2014). Extremely high levels of lactic acid are generally known to be lethal, but our results show that subclinical elevation of lactic acid levels could play a role in cardiometabolic disease as well. Importantly, high lactate levels, as a product of oxidation of pyruvic acid, are indicative of increased anaerobic metabolism, and increased oxidative stress.

Lysophosphatidylcholine(18:2) levels were lower in the univariate regression analysis in individuals with metabolic syndrome score as compared to controls, but we found no consistent relation between lysophosphatidylcholines as a chemical class and the metabolic syndrome score in the OPLS model. Lysophosphatidylcholines play a pivotal role in oxidized LDL cholesterol, and are found to directly affect progression of atherosclerosis through multiple biological pathways including inflammatory processes (Aiyar et al. 2007; Lusis 2000). Previously, lower concentrations of lysophosphatidylcholines have been observed in obesity and type 2 diabetes mellitus (Barber et al. 2012), and might directly affect insulin resistance state (Motley et al. 2002). Furthermore, an inverse relationship between serum lysophosphatidylcholines and vascular damage and heart rate was observed in patients with atherosclerosis (Paapstel et al. 2018).

In the lipid profiling analysis, we identified predominantly triglycerides to be correlated with the metabolic syndrome. Although not unexpected given the triglyceride subcomponent, we observed the odd-chain triacylglycerol (53:2/3), which originates mainly from food, to be negatively correlated to the metabolic syndrome as well as with several of its subcomponents in the OPLS analysis. In addition, multiple ceramides were positively correlated with the metabolic syndrome and a number of its components. A positive relationship between ceramide levels and insulin resistance has been found previously (Blachnio-Zabielska et al. 2012). Interestingly, in literature, ceramides are described to be important mediators of oxidative stress in apoptosis signalling (Andrieu-Abadie et al. 2001). Furthermore, a number of ether-bound phosphatidylcholines were negatively correlated with the metabolic syndrome. Interestingly, this biochemical class is associated with decreased oxidative stress levels and slows the ageing process (Hung et al. 2001).

The main strength of the present study was to investigate the connection between metabolite and lipid profile and the metabolic syndrome using platforms enabling detection of many compounds not frequently investigated in unstandardized human population studies. However, the use of an unstandardized human population likely resulted in an increased variability in the data as a consequence of factors like lifestyle and disease heterogeneity. This increased variability in the data is likely the cause of the limited separation in the PCA score plots. Nevertheless, using this approach, we were able to provide (novel) insights that could be used in future population and experimental studies. Validity of the results was confirmed by checking significance of the obtained OPLS models, application of univariate analysis and by putting the results into biological context based on the available scientific literature. Still, since metabolomics/lipidomics is an exploratory approach, with usually limited amount of samples included in the hypothesis generating study (as was the case also for present study). the described findings require verification in the independent cohorts. Given the observational nature of the data, no causality of our research findings can be inferred. Furthermore, due to the design of the study, causality cannot be determined (e.g., the altered metabolite/lipid concentration could be either the cause as well as the consequence of the metabolic syndrome condition).

In summary, within this first combined metabolomics and lipidomics study on the metabolic syndrome, we identified several metabolites and lipids to be connected to the metabolic syndrome, which could be of interest for further research in the field of cardiometabolic disease biology. Interestingly, several of the different biochemical pathways that we identified in relation to the metabolic syndrome have been previously found to be connected to the regulation of oxidative stress. Future studies are however required to further elucidate our research findings.

## Electronic supplementary material

Below is the link to the electronic supplementary material.

Supplementary material 1 (DOCX 18 KB)

Supplementary material 2 (XLSX 84 KB)

Supplementary material 3 (DOCX 112 KB)
