# Supplementary material for: Metabolomic and lipidomic assessment of the metabolic syndrome in Dutch middle-aged individuals reveals novel biological signatures separating health and disease
Source: Metabolomics. 2019 Feb 12;15(2):23. doi: 10.1007/s11306-019-1484-7 (PMC6373335; doi:10.1007/s11306-019-1484-7)
Supplement: Supplementary file 1 — Supplementary material 1 (DOCX 18 KB) [file 11306_2019_1484_MOESM1_ESM.docx]

**Metabolomic and lipidomic assessment of the metabolic syndrome in Dutch middle-aged individuals reveals novel biological signatures separating health and disease**

Izabella Surowiec^1,*^, Raymond Noordam^1,2,*^, Kate Bennett^1^, Marian Beekman^3^, P Eline Slagboom^3^, Torbjörn Lundstedt^1,#^, Diana van Heemst^2,#^

1. AcureOmics AB, Umeå, Sweden
2. Department of Internal Medicine, Section of Gerontology and Geriatrics, Leiden University Medical Center, Leiden, the Netherlands
3. Department of Medical Statistics and Bioinformatics, Section of Molecular Epidemiology, Leiden University Medical Center, Leiden, the Netherlands

* Shared-first author, # Shared-last author

**Supplementary Methods**

*Derivatization of samples before GC-MS analysis*

A 30 µl of methoxyamine (15 µg/µl) in pyridine was added to the dried extract and the resultant mixture was shaken vigorously for 10 min. Methoxymation was performed at room temperature for 16 h, followed by the addition of 30 µl MSTFA with 1% TMCS to each sample (brief vortex). Samples were left at room temperature for 1 h to allow silylation to occur, followed by the addition of 30 µl heptane (containing 15 ng/µl methyl stearate as an internal standard) and a brief vortex for 10 s.

*GC-MS metabolomics analysis*

A volume of 1 µl of each derivatzed sample was injected splitless by a CTC Combi Pal autosampler (CTC Analytics AG, Zwingen, Switzerland) into an Agilent 7890 GC equipped with a 10 m x 0.18 mm i.d. fused-silica capillary column chemically bonded with 0.25-um DB 5-MS stationary phase (J&W Scientific Folsom, CA). The injector temperature was set to 260^o^C. Helium was used as the carrier gas at a constant flow rate of 1 mL min-1 through the column. For every analysis, the purge time was set to 75 s at a purge flow rate of 20 mL/min and an equilibrium time of 1 min. The column temperature was held initially at 70^o^C for 2 min, then increased to 320^o^C at a rate of 40^o^C/min, where it was held for 2 min. The column effluent was introduced into the ion source of a Pegasus III time-of-flight mass spectrometer (Leco Corp., St Joseph, MI, USA). The ion source and transfer line temperatures were set to 200^o^C and 250^o^C, respectively. Ions were generated by a 70 eV electron beam at a current of 2.0 mA. Masses were acquired in the mass range 50-800 m/z at a rate of 20 spectra/s. The acceleration voltage was turned on after a solvent delay of 150 s. The detector voltage was 1670 V.

An alkane series (C10-C40) was run together with all samples to enable calculation of retention indices (RI) of the compounds.

*LC-MS metabolomics analysis*

Aliquots of samples (2 µL) were injected into an Agilent UPLC system (Infinity 1290) equipped with a UPLC column (Acquity HSS T3, 2.1 x 50 mm, 1.8 µm C18 in combination with a 2.1 mm x 5 mm, 1.8 µm VanGuard precolumn (Waters Corporation, Milford, MA, USA). The UPLC system was coupled to an Agilent 6550 iFunnel Jet stream electrospray ion source Accurate-Mass QTOFMSMS (Agilent Technologies, Santa Clara, CA, USA). The mobile phases used were MilliQ water containing 0.1 % formic acid (A) and a mixtures of 75:25 acetonitrile:2-propanol with 0.1 % formic acid (B). The following gradient was used: 0.1 - 10 % B for 2 min at a flow rate of 0.5 mL/min, then B was increased to 99 % during 5 min and held at 99 % for 2 min, B was then decreased to 0.1 % during 0.3 min and the flow-rate was increased to 0.8 mL/min for 0.5 min; these conditions were maintained for 0.9 min after which the flow-rate was reduced to 0.5 mL/min for 0.1 min before injection of the subsequent sample. Column oven temperature was held at 40°C whilst samples were kept at 5°C in the auto sampler at. Analysis was initially performed in the positive mode for all samples and subsequently in the negative mode using a second injection of each sample. Mass spectrometry parameters, with exception of the capillary voltage, were kept identical between the modes

A reference interface was connected to ensure accurate mass measurements; the reference ions purine (4 µM) and HP-0921 (Hexakis(1H, 1H, 3H-tetrafluoropropoxy)phosphazine) (1 µM) both purchased from Agilent Technologies (Santa Clara, CA, USA) were infused directly into the mass spectrometer at a flow rate of 0.05 mL/min for internal calibration, and the monitored ions were purine m/z 121.05 and m/z 119.03632; HP-0921 m/z 922.0098 and m/z 966.000725 for positive and negative mode, respectively. The gas temperature was set to 150°C, the drying gas flow to 16 L/min and the nebulizer pressure 35 psi. The sheath gas temp was set to 350°C and the sheath gas flow to 11 L/min. The capillary voltage was set to 4000 V in positive ion mode, and to 4500 V in negative ion mode. The nozzle voltage was 300 V. The “fragmentor” voltage was 380 V, the skimmer 45 V and the OCT 1 RF Vpp 750 V. The collision energy was set to 0 V. The m/z range was 70 - 1700, and data were collected in centroid mode with an acquisition rate of 4 scans/s.

*LC-MS lipidomics analysis*

An aliquot (2 µL) of the sample was injected into an Agilent UPLC system (Infinity 1290) equipped with a UPLC column (Acquity CSH, 2.1 x 50 mm, 1.7 µm C18 in combination with a 2.1 mm x 5 mm, 1.7 µm VanGuard CSH precolumn; Waters Corporation, Milford, MA, USA). The UPLC system was coupled to an Agilent 6540 iFunnel Jet stream electrospray ion source QTOFMSMS (Agilent Technologies, Santa Clara, CA, USA). Mobile phases used were 60:40 ACN:water + 10 mM ammonium formate + 0.1% formic acid (A) and 89.1:10.5:0.4 IPA:ACN:water + 10 mM ammonium formate + 0.1% formic acid (B). The following gradient was used: 0.0 - 15 % B at a flow rate of 0.5 mL/min, then B was increased to 30 % during 1.2 min, then to 55% during 0.3 min and held at 55 % for 3.5 min, then to 72% during 2 min, to 85% during 2.5 min and to 100% during 0.5 min where was held at 0.5 ml/min flow rate for additional 2 min after which the flow rate was increased to 5ml/min and kept for 0.5 min to wash the injection valve. After 12 min data was no longer acquired by the mass spectrometer. The column was equilibrated with 15% B for 1.5 min at a flow rate of 0.5 ml/min before the next sample was injected. The column oven temperature was held at 65°C and samples were kept in auto sampler at 10°C. Analysis was performed in the positive ion mode. A reference interface was connected to facilitate accurate mass measurements; both of the reference ions (purine (4 µM) and HP-0921 (Hexakis(1H, 1H, 3H-tetrafluoropropoxy)phosphazine) (1 µM) were purchased from Agilent Technologies (Santa Clara, CA, USA)) were infused directly into the MS at a flow rate of 0.08 mL/min for internal calibration, and the monitored ions were purine m/z 121.05 and m/z 119.03632; HP-0921 m/z 922.0098 and m/z 966.000725 for positive and negative mode, respectively. The gas temperature was set to 300°C, the drying gas flow to 8 L/min and the nebulizer pressure 40 psi. The sheath gas temp was set to 350°C and the sheath gas flow to 11 L/min. The capillary voltage was set to 4000 V in positive ion mode. The nozzle voltage was 0 V. The fragmentor voltage was 100 V, the skimmer 45 V and the OCT 1 RF Vpp 750 V. The collision energy was set to 0 V. The m/z range was 70 - 1700, and data were collected in centroid mode with an acquisition rate of 4 scans/s.
