# Supplementary material for: Metabolomic and lipidomic assessment of the metabolic syndrome in Dutch middle-aged individuals reveals novel biological signatures separating health and disease
Source: Metabolomics. 2019 Feb 12;15(2):23. doi: 10.1007/s11306-019-1484-7 (PMC6373335; doi:10.1007/s11306-019-1484-7)
Supplement: Supplementary file 3 — Supplementary material 3 (DOCX 112 KB) [file 11306_2019_1484_MOESM3_ESM.docx]

**Metabolomic and lipidomic assessment of the metabolic syndrome in Dutch middle-aged individuals reveals novel biological signatures separating health and disease**

Izabella Surowiec^1,*^, Raymond Noordam^1,2,*^, Kate Bennett^1^, Marian Beekman^3^, P Eline Slagboom^3^, Torbjörn Lundstedt^1,#^, Diana van Heemst^2,#^

1. AcureOmics AB, Umeå, Sweden
2. Department of Internal Medicine, Section of Gerontology and Geriatrics, Leiden University Medical Center, Leiden, the Netherlands
3. Department of Medical Statistics and Bioinformatics, Section of Molecular Epidemiology, Leiden University Medical Center, Leiden, the Netherlands

* Shared-first author, # Shared-last author


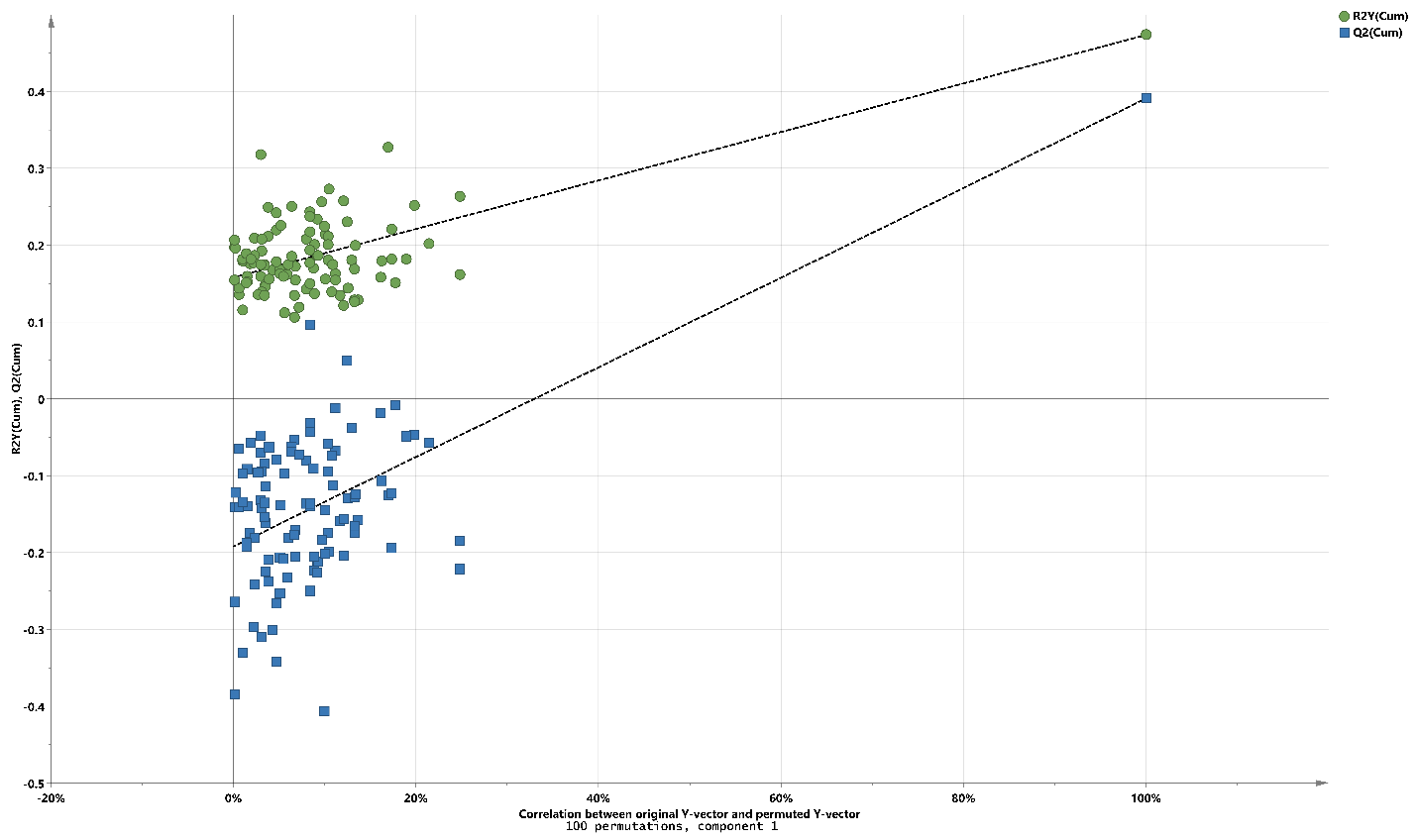


**Supplementary Figure 1**. OPLS permutation plot for the metabolomics data set displaying the correlation coefficient between the original Y variable (metabolic syndrome score) and the permuted Y variable on the x-axis versus the cumulative R^2^Y and Q^2^ on the y-axis, with the regression line between them. The intercept is the measure of the over fit. The Y-axis intercept is below 0.3 for R^2^Y and below 0.05 for Q^2^.


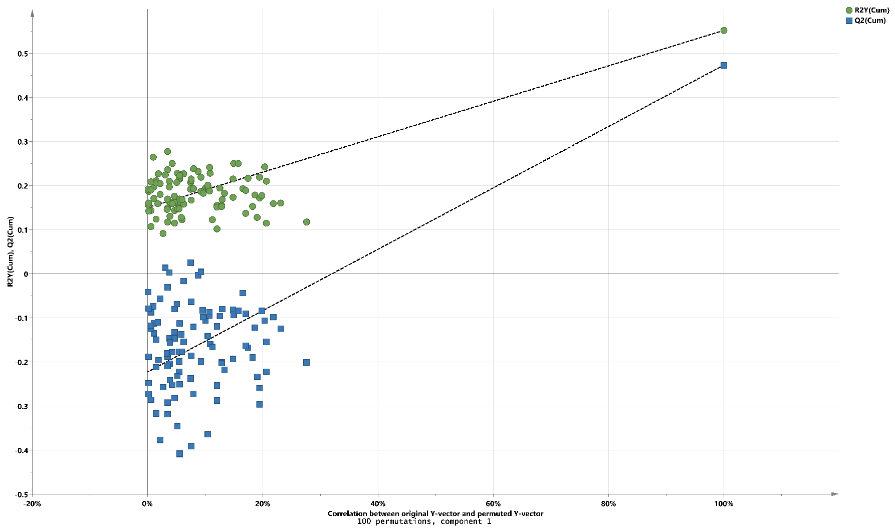


**Supplementary Figure 2**. OPLS permutation plot for the lipidomics data set displaying the correlation coefficients between the original Y variable (metabolic syndrome score) and the permuted Y variable on the x-axis versus the cumulative R^2^Y and Q^2^ on the y-axis, with the regression line between them. The intercept is the measure of the over fit. The Y-axis intercept below 0.3 for R^2^Y and below 0.05 for Q^2^.
